# Poly (acrylic acid sodium) grafted carboxymethyl cellulose as a high performance polymer binder for silicon anode in lithium ion batteries

**DOI:** 10.1038/srep19583

**Published:** 2016-01-20

**Authors:** Liangming Wei, Changxin Chen, Zhongyu Hou, Hao Wei

**Affiliations:** 1 Key Laboratory for Thin Film and Microfabrication of the Ministry of Education, Department of Microelectronics and Nanoscience, School of Electronic Information and Electrical Engineering, Shanghai Jiao Tong University, 800 Dongchuan Road, Shanghai, 200240, China

## Abstract

The design of novel binder systems is required for the high capacity silicon (Si) anodes which usually undergo huge volume change during the charge/discharge cycling. Here, we introduce a poly (acrylic acid sodium)-grafted-carboxymethyl cellulose (NaPAA-*g*-CMC) copolymer as an excellent binder for Si anode in lithium ion batteries (LIBs). The NaPAA-*g*-CMC copolymer was prepared via a free radical graft polymerization method by using CMC and acrylic acid as precursors. Unlike the linear, one-dimensional binders, the NaPAA-*g*-CMC copolymer binder is expected to present multi-point interaction with Si surface, resulting in enhanced binding ability with Si particles as well as with the copper (Cu) current collectors, and building a stable solid electrolyte interface (SEI) layer on the Si surface. The NaPAA-*g*-CMC based Si anode shows much better cycle stability and higher coulombic efficiency than those made with the well-known linear polymeric binders such as CMC and NaPPA.

The development of rechargeable lithium batteries with high energy density requires finding alternative anode materials with higher charge storage capacity than that of traditional graphite anode materials^1^,^2^. Silicon (Si) has been proposed as one of the most promising anode materials for next generation lithium-ion batteries due to its high theoretical lithium storage capacity (4200 mA h g^−1^ for Li_22_Si_5_) which is more than about 10 times that of commercial graphite (372 mA h g^−1^)^3^,^4^,^5^. The major drawback of using Si as anode for lithium ion batteries is the large volume change (>300%) during charge/discharge cycling. This large volume change could cause the pulverization of the Si particles and electrical disconnection, thereby leading to fast capacity decay and short cycle life^6^,^7^,^8^,^9^,^10^,^11^. Tremendous effect has been made to overcome this problem^12^, including controlled voltage^13^,^14^, design of novel nanostructured Si materials and synthesis of Si-based composites^15^,^16^,^17^,^18^,^19^,^20^,^21^,^22^,^23^,^24^,^25^,^26^,^27^,^28^ *et al.* Among various strategies investigated, a robust polymeric binder has turned out a promising means to maintain both electrode integrity and electric conductivity for Si-based anode^29^,^30^.

Polymeric binders hold active materials and conducting carbon additives together, and adhere them onto metal current collectors. The polymeric binder’ properties have close relationship with the electrochemical performance of the batteries, including the cycle life and irreversible capacity loss. Many studies have confirmed that the functional polymers containing carboxyl groups, such as carboxymethyl cellulose (CMC) and poly(acrylic acid sodium) (NaPAA) are better binders for Si-based anodes than non-functional polymeric binders such as poly(vinylidene fluoride) (PVDF) and styrene-butadiene rubber (SBR)^31^,^32^. The hydrogen bonding and/or covalent chemical bonds between the carboxyl groups of the functional binders and the hydroxyl groups on the Si surface are proposed to favor the long cycle stability of Si anodes^29^. A high young’ s modulus alginate binder containing higher content of carboxyl groups shows better electrochemical performance for Si-based anodes^30^. Moreover, grafting multi-functional groups such as dopamine onto PAA or introduction of methyl benzoate ester, triethyleneoxide monomethyl ether and fluorenone to conductive polymers further improves the performance of silicon anodes^33^,^34^,^35^. Nonetheless, the one-dimensional (1D), linear chain nature of these polymers limits their multi-point interaction with Si particles^36^. As a result, the polymer chains tend to slide from Si surface during cycling. Therefore, it is desirable to develop three-dimensional polymer networks, such as interpenetrated PAA/polyvinyl alcohol gel^31^, crosslinked CMC-PAA^32^ and hyperbranched β-cyclodextrin^36^ to improve the interaction between Si and binders, thus enhancing electrochemical performance of the batteries. Although a significant progress has been achieved in the Si anode by the use of these polymeric binders, the development of novel binder systems is still required for the practical application of the high capacity Si anodes in lithium ion batteries.

Herein, we develop a novel NaPAA grafted-CMC polymeric binder (NaPAA-*g*-CMC) for Si anodes through free radical graft polymerization of acrylic acid onto the linear CMC backbone. The NaPAA-*g*-CMC binders with branched structure is believed to present multi-point interaction with Si surface, resulting in enhanced binding ability with Si particles as well as with the copper (Cu) current collectors, and building a stable SEI layer on the Si surface. The PAA-*g*-CMC based Si anode exhibits significantly improved cycling performance when compared with those with well-known linear polymer binders such as NaPAA and CMC.

## Methods

### NaPAA-*g*-CMC preparation

A 1 g of CMC power was dissolved in 50 mL distilled water form a transparent sticky solution, and then the solution was put in a 250 mL three-necked flask equipped with a mechanical stirrer. After being purged with nitrogen for 6 h to remove oxygen, 2.5 ml of acrylic acid monomer and the initiator (NH_4_)_2_S_2_O_8_/NaHSO_3_, 0.1:0.03 g) were added. The solution was then heated to 55 °C under continuous stirring with rpm of 270 for 2h to produce PAA-*g*-CMC. The nitrogen atmosphere was maintained throughout the reaction process. Finally, the PAA-*g*-CMC was neutralized by sodium hydroxide solution to PH 6.

### Electrochemical Experiments

CR2016-type coin cells were assembled in an argon-filled glove box with oxygen and water contents less than 1 ppm, using lithium-foil as counter electrode and a polymer as a separator. The electrolyte was 1M LiPF_6_ dissolved in the mixture of dimethyl carbonate (DMC) and ethylene carbonate (EC) (1:1 in volume ratio), plus 10 wt% fluoroethylene carbonate (FEC) as additive. The working electrode consisted of 60 wt% Si (the Si nanoparticles was obtained from Alfa Aesar, and the diameter ranged from 50–100 nm. The SEM, TEM and the particle size analysis of the pristine Si nanoparticles were shown in Fig. S1, Supporting Information), 20 wt% conducting agent (Super P) and 20 wt% binders. The loading weight of pure Si on the copper foil is about 0.45 mg cm^−2^. The electrochemical performances were tested on a LAND battery test system (Wuhan, China) at room temperature, in the voltage range of 0.01–1.2 V.

### Measurement

The morphologies of the samples were characterized by field emission scanning electron microscopy (SEM, Carl Zeiss Ultra 55). Fourier transform infrared (FTIR) spectra were measured on Perkin-Elmer 70 using KBr pieces. The transmission electron microscopy (TEM) images were obtained from JEOL 2100. The particle size was measured from Brookhaven BI-90 plus.

For 180° peel test, the electrodes without Super P were first prepared. The mass ratio of Si : binder was 4: 1, and the thickness of each electrode materials was about 50 μm. Before the peel test, the electrode film was cut into a 30 mm wide and 60 mm long specimen, and the KingTiger tape was then attached to the specimen. This tape was peeled off using a high-precision material mechanical testing machine (Shanghai Hengyi Testing Machine Company, Shanghai, China). The peel strength was monitored while the force-displacement plot was recorded.

## Results

### Synthesis and characterization of NaPAA-*g*-CMC binder

**Figure 1:**
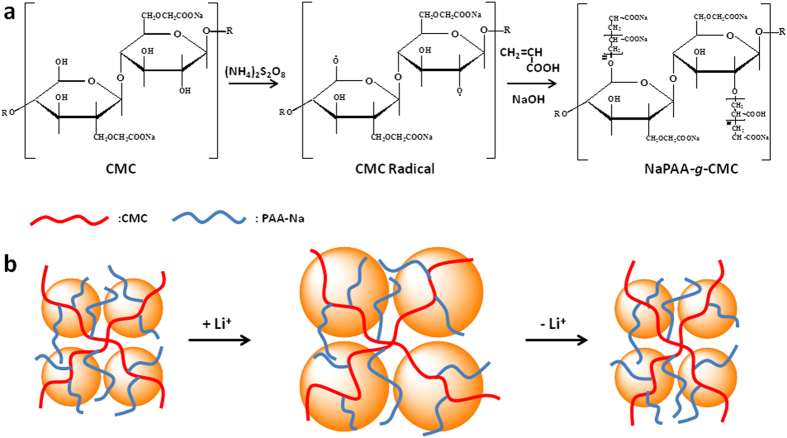
(**a**) Synthetic scheme for covalent graft of NaPAA onto CMC backbone; (**b**) possible working mechanism of NaPAA-*g*-CMC binder to accommodate the lager volume change of Si particles during cycling.

**Figure 2:**
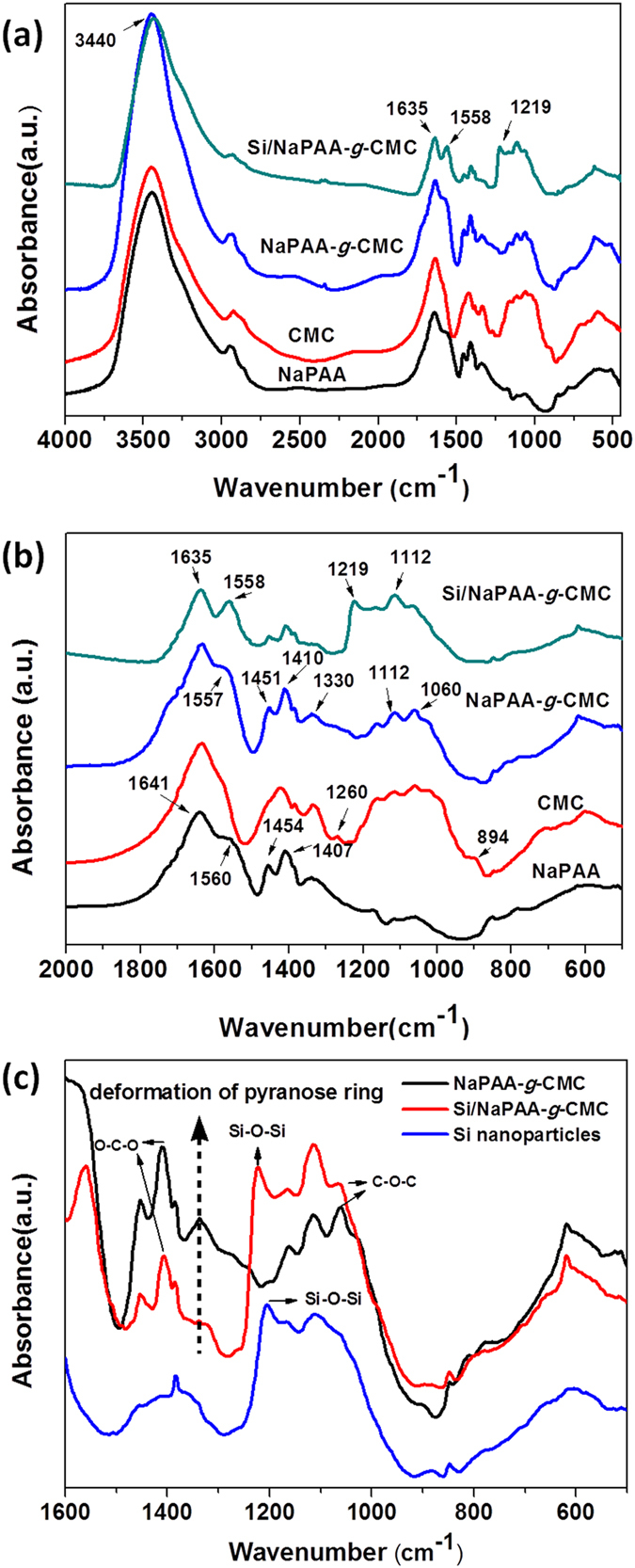
(**a**) FTIR spectra of NaPAA, CMC, NaPAA-*g*-CMC binders and Si/NaPPA-*g*-CMC mixture; (**b,c**) highlighted some portions of the FTIR spectra.

The NaPAA-*g*-CMC polymeric binders were synthesized via free radical graft polymerization by using CMC and acrylic acid as precursors and persulphate as a radical initiator. The mechanism for the formation of NaPAA-*g*-CMC is shown in Fig. 1. The persulphate initiator decomposed to generate anionic radical (KSO_4._^−^) upon heating. It is well-known that this anionic radical can abstracts hydrogen from the hydroxyl group of CMC to form macro-radicals which initiated the acrylic monomer polymerization, leading to formation of the grafted chains^37^,^38^,^39^. FTIR spectra were used to characterize the NaPAA, CMC, NaPAA-*g*-CMC and Si/NaPAA-*g*-CMC samples (Fig. 2). After grafting polymerization, the absorption bands at 1260 (the in-plane bending vibration of -OH) and 894 cm^−1^ (the symmetric stretching vibration of C-O) almost disappeared when compared with pure CMC, whereas two new absorption bands at 1557 and 1451 cm^−1^ ascribed to the COO^−^ groups of NaPAA appeared in the spectrum of NaPAA-*g*-CMC. These results evidenced graft of NaPAA onto CMC^38^.

The FTIR spectroscopy was also used to characterize the interaction between the NaPAA-*g*-CMC binder and Si powder. The NaPAA-*g*-CMC binder exhibits an absorption peak at 1330 cm^−1^ assigned to pyranose-ring deformation vibration^40^. Such peak intensity decreases significantly after mixture with Si nanoparticles (Fig. 2c). This decrease confirms a strong interaction between the NaPAA-*g*-CMC binders and the Si nanoparticles^30^, which might stem from the strong hydrogen bonding between NaPAA-*g*-CMC carboxylic moieties and SiO_2_ surface on Si nanoparticles (the formation of SiO_2_ surface on Si was confirmed by FTIR as shown in Fig. S2). The strong interaction between the polymeric binder and the Si surface is previously identified as the key factor affecting the stability of the Si-based electrodes^30^,^31^,^32^,^33^,^34^.

### Electrochemical performance of Si anodes

**Figure 3:**
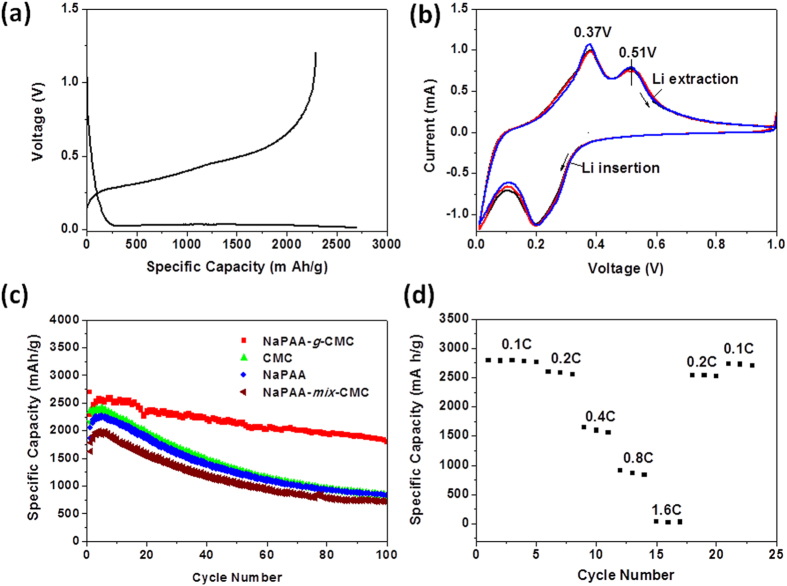
(**a**) The galvanostatic voltage profile of the Si anode made with NaPPA-*g*-CMC binder in the first charge/discharge cycle; (**b**) the cyclic voltammetry spectra of NaPPA-*g*-CMC based Si anodes; (**c**) the cycling performance of Si electrodes made with PAA, CMC, NaPAA-*g*-CMC and the simple mixture of NaPAA/CMC binders; (**d**) cycling performance of the Si electrodes made with NaPAA-*g*-CMC at different current density from 0.1C (420 mAg^−1^) to 1.6C (6.7 A g^−1^).

The electrochemical performance of the Si anodes was examined using galvanostatic cycling at room temperature using a coin-type half-cell which was constructed using Si as the anode, lithium foil as a counter electrode and a polymer as a separator. The Si electrode consisted of 60 wt% Si nanoparticles, 20 wt% conducting agent (Super P) and 20 wt% binder. The cell was charged and discharged between 0.01–1.2 V at a current density of 0.2C (840 mA g^−1^). As shown in the voltage profile of the Si electrode made with NaPAA-*g*-CMC binder, the discharge curve displays a long flat plateau below 0.25 V (Fig. 3a), which corresponds to the Li-alloying process of crystalline Si to form amorphous Li_x_Si phase^3^,^5^. Cyclic voltammetry (CV, Fig. 3b) measurement shows two delithiation peaks at 0.37 V and 0.51 V and a lithiation peak at 0.2 V^30^. The three reproductive CV cycles indicate a good stability for NaPAA-*g*-CMC-based electrode.

Figure 3c comparatively exhibits the electrochemical performance of Si electrodes made with PAA, CMC, NaPAA-*g*-CMC and the simple mixture of NaPAA/CMC binders (denoted as Si_PAA_, Si_CMC_ and Si_NaPAA-*g*-CMC_ and Si_NaPAA-*mix*-CMC,_ respectively). A reversible Li-extraction capacity of 2290 mA h g^−1^ was obtained in the first cycle for Si _NaPAA-*g*-CMC_ with Si loading of 0.45 mg cm^−2^. After 100 cycles, the capacity remained 1816 mA h g^−1^, about 79.3% capacity retention. We also found that when the mass loading of Si decrease to 0.19 mg cm^−2^, higher capacity retention of 87% can be obtained after 100 cycles (Fig. S3). In contrast, Si_PAA_, Si_CMC_ and Si_NaPAA-*mix*-CMC_ show fast capacity fading. The capacity retention was only 39%, 45.5%, 43% for Si_CMC_, Si _PAA_ and Si _NaPAA-*mix*-CMC_, respectively, after the 100th cycle. The remarkably improvement in cycle stability for Si_NaPAA-*g*-CMC_ indicates the significance of covalent graft of PAA to CMC backbone.

The coulombic efficiency (CE) of Si_NaPAA-*g*-CMC_ was 84% at the first cycle, and it increased to 99.4% at the 100th cycle, leading to an average CE of 98.4% in the cycling range of 1–100 (Fig. S4). In contrast, the average CE was 96.8%, 97%, 96.9% for CMC, PAA and Si_NaPAA-*mix*-CMC_, respectively. The enhanced CE for Si_NaPAA-*g*-CMC_ suggests the NaPAA-*g*-CMC binder helps building a more stable SEI layer on the Si surface. It is desirable to further increase CEs of Si_NaPAA-*g*-CMC_ for practical full-cell application by surface treatments and electrolyte modifications^3^,^36^. Figure 3d demonstrated rate performance of Si_NaPAA-*g*-CMC_. A reversible capacity of ~900 mA h g^−1^ at current density of 3.36 Ag^−1^ (0.8 C) can be achieved. Importantly, after charge/discharge measurement at high current density of 6.72 Ag^−1^(1.6 C), the capacity can fully recover to the initial value. Clearly, Si_NaPAA-*g*-CMC_ demonstrated high reversibility.

**Figure 4:**
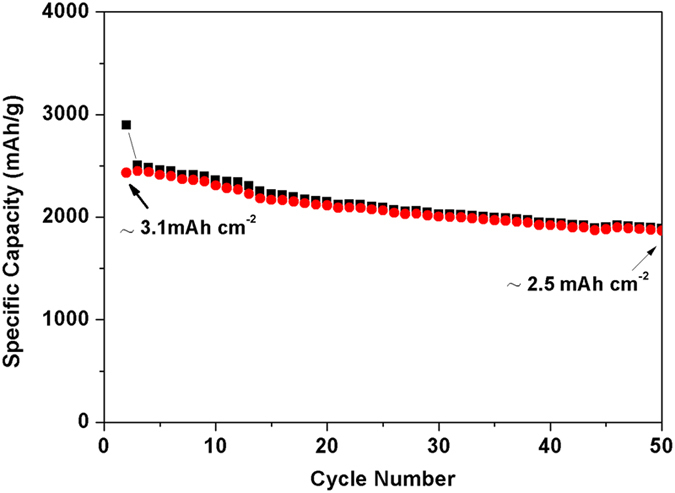
The cycling performance of Si electrodes with Si mass loading of 1.3 mg cm^−2^ using NaPAA-*g*-CMC as binders.

Besides the specific capacity (mAh g^−1^), the area capacity of the electrodes (mAh cm^−2^) is widely used to evalute the electrode performance in the practical application. Obviously, obtaining high area capacity requires high mass loading of active materials on the electrodes. Generally, increasing the Si mass loading will lead to decrease in the specific capacity and fast capacity fading during cycling. In this work, the Si_NaPAA-*g*-CMC_ anode with a high mass loading of 1.3 mg cm^−2^ still exhibits good cycling performance (Fig. 4). After 50 cycles, the capacity remained 1880 mA h g^−1^, about 80% capacity retention, and the area capacity can reach up to 2.5 mAh cm^−2^.

When synthesis of NaPAA-*g*-CMC, around 3% of persulfacte was used as initiators. To investigate the effect of the initiators on the electrochemical performance of the silicon anode, we precipitated NaPAA-*g*-CMC from the aqueous solution by ethanol (the persulfacte initiators cannot be precipitated from the aqueous solution), and then used the obtained NaPAA-*g*-CMC as binder for the Si anodes. We found that removal of the initiators increased the capacity of the Si anode, but little effected the cycle stability (about 76% capacity retention after 100 cycle with Si loading of 0.48 mg cm^−2^, Fig. S5).

### Binding ability of binders and morphology of the cycled Si anode

**Figure 5:**
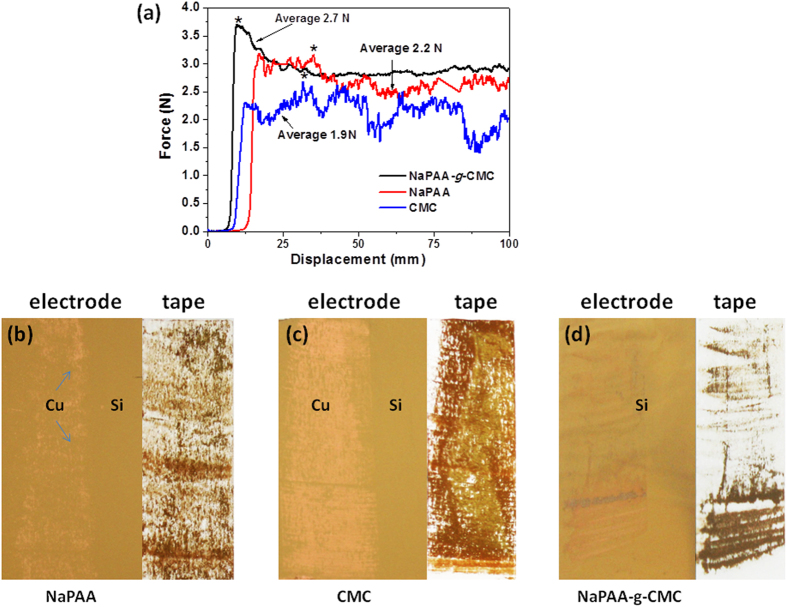
(**a**) The 180^o^ peeling tests for the Si electrode with different binders; (**b**) the optical images of the electrode and tape surfaces after the peel test. When NaPAA-*g*-CMC was used as binder, most of electrode materials were kept on the surface of Cu foil.

It is known that the PAA and CMC binders form strong hydrogen bonding between the hydroxyl groups on the Si surface and the carboxyl groups of the binders^29^. This strong interaction makes the Si electrode provide better cycle performance than the Si electrodes with PVDF binders which bind to Si surface via weak val der Waals interaction. Unlike the linear structured PAA and CMC, the branched Si_NaPAA-*g*-CMC_ binder was proposed to present increased number of contacts between the binders and Si particles, leading to enhanced binding ability with Si particles as well as Cu foil. To assess the adhesion of the binder in Si anodes, the 180^o^ peeling test was carried out. The force-displacement curves of the Si electrodes as shown in Fig. 5 indicate the adhesion between Si, binder and Cu foil. It can be seen that the PAA, CMC and NaPAA-*g*-CMC binders exhibits distinctive behavior. The initial peeling forces were 2.3, 3.2 and 3.7 N for CMC, PAA and NaPAA-*g*-CMC, respectively. The average peeling force of NaPAA-*g*-CMC (2.7 N) is also higher than that of PAA (2.2 N) and CMC (1.9 N) binders (Fig. 5a). This enhanced adhesion for NaPAA-*g*-CMC evidenced that the branched NaPAA-*g*-CMC binder imparted higher mechanical stability to the Si electrode than the linear structured PAA and CMC binders. Moreover, the optical images (Fig. 5b–d) show that when PAA or CMC was used as binders, a large portion of Si-base electrode materials was peeled off from the Cu foil and adhered on the tape side after the peeling tests, whereas most of electrode materials were still kept on the surface of Cu foil when NaPAA-*g*-CMC was used as binder. This result further confirmed that NaPAA-*g*-CMC binder enhanced the adhesion between the Si particles as well as between the Si particles and Cu foils. Such strong adhesion is crucial for preservation of the Si electrode structure during charge and discharge cycles^33^. It should be mentioned that the peeling force for Si _NaPAA-*g*-CMC_ mainly characterizes the binding ability between the adhesive tape and the electrode surface because few electrode materials was peeled from the Cu foil (Fig. 5d). We expect that if a adhesive tape with adhesive force large enough to peel the electrode materials from the Cu foil (just like in the case of Si_NaPAA_ or Si_CMC_) was used, an average peel force large than 2.7 N for Si _NaPAA-*g*-CMC_ could have been observed.

**Figure 6:**
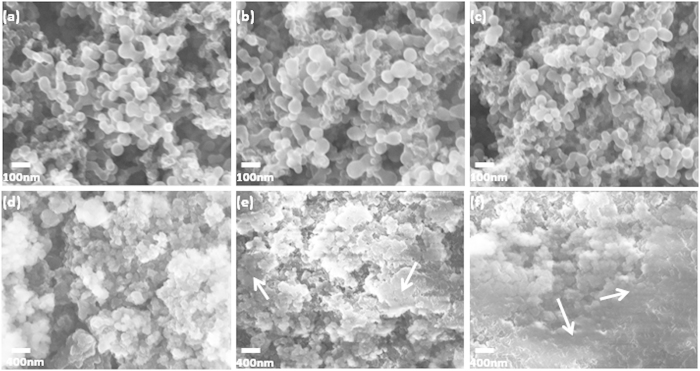
(**a**) SEM images of silicon electrodes with different binders after 100 deep cycles(cycle potential window from 0.01 to 1.2 V *vs* Li/Li^+^).

In order to better understand the improved electrochemical performance for NaPAA-*g*-CMC, the scanning electron microscopy (SEM) was used to characterize the surface morphologies of Si electron films with different binders before and after 100 deep cycles. Figure 6a–c show that the Si_PAA-*g*-CMC_, Si_PAA_ and Si_CMC_ samples exhibit uniform particle distribution and surface porosities before cycling. The obvious difference in the morphology was observed for these samples after 100 deep cycles. A smooth and thick solid electrolyte interface (SEI) layer was found to cover Si nanoparticles in the cycled Si_PAA_ and Si_CMC_ samples. This smooth and thick SEI layer is believed to be due to pulverization of Si particles resulting from the large volume change during cycling and subsequent growth of additional SEI on these newly exposed Si surface^1^,^31^. This SEI layer filled the porosity of electrodes, resulting in smooth topography on the electrode surface. In contrast, in the cycled Si_NaPAA-*g*-CMC_ sample, the individual Si particle with spherical shape was visible and porous structure of the electrode remained. Thus, a thinner SEI layer than those forming on Si_CMC_ or Si_NaPAA_ is speculated to form in Si _PAA-*g*-CMC_ after repeated cycles. The formation of the thin SEI might result from formation of a better protective layer by NaPAA-*g*-CMC binder on the Si nanoparticle surface; furthermore, the enhanced binding ability between the Si nanoparticles, NaPAA-*g*-CMC binder and the Cu foil might make Si nanoparticles possible to accommodate large volume change during cycling, and should assist in building a stable SEI layer on Si particles^33^,^36^. Such stable SEI is believed to play an important role in enhancing cycle life and improve CE of the Si based anodes^30^,^31^.

## Discussion

The high electrochemical performance of Si_NaPAA-*g*-CMC_ could be explained by the different binding mechanism when compared to PAA and CMC binders. The PAA and CMC binders with a linear and one dimensional structure present only one point or linear contact between Si particles and binders^36^, and thus they are easily detached from the surface of the Si particles when the Si particles repeatedly expanded and shrank during lithiation-delithiation cycling. In contrast, the NaPAA-*g*-CMC binder with branched structure might easily form 3D polymer network around the Si particles via hydrogen bonding and van der Waals interaction (Fig. 1b). This polymer network could present multi-point contact with the surface of Si, resulting in enhanced binding ability with the Si particles. This multiple point contact is favorable for preservation of the electrode stability during cycling even if a large volume change of Si particles takes place. There are two possible mechanisms to preserve the electrode stability during cycling when using NaPAA-*g*-CMC as binder: when one branch of the polymer loses the contact on one spot of the Si surface, another branch of the polymer might still keep the contact with the Si particles; the polymer binders, which have lost the contact with Si on one point during the delithiation, might recover the contact at another point in the 3D polymer network during the following cycles^36^, and thus, an highly enhanced cycling performance was obtained.

## Conclusion

We have developed a NaPAA-*g*-CMC copolymer as an excellent binder for Si anode in LIBs by using an *in-situ* grafting polymerization method. This copolymer binder exhibits strong binding ability with Si nanparticles was well as with the Cu current collectors, and also assisted in building a stable SEI layer on the Si surface. The resulting NaPAA-*g*-CMC based anode shows high capacity, excellent cycle stability and high coulombic efficiency. The presence of multi-point interaction with Si surface might account for the enhanced electrochemical performance for NaPAA-*g*-CMC. The NaPAA-*g*-CMC binder with the advantages of low cost, easy synthesis and eco-friendliness provided a competitive solution for high capacity Si anodes, and also can be applied to other high capacity electrode materials other than Si anode.

## Additional Information

**How to cite this article**: Wei, L. *et al.* Poly (acrylic acid sodium) grafted carboxymethyl cellulose as a high performance polymer binder for silicon anode in lithium ion batteries. *Sci. Rep.* **6**, 19583; doi: 10.1038/srep19583 (2016).

## Supplementary Material

Supplementary Information
