# Supplementary material for: Poly (acrylic acid sodium) grafted carboxymethyl cellulose as a high performance polymer binder for silicon anode in lithium ion batteries
Source: Sci Rep. 2016 Jan 20;6:19583. doi: 10.1038/srep19583 (PMC4726210; doi:10.1038/srep19583)
Supplement: Supplementary Information [file srep19583-s1.pdf]

Supplementary Information

Poly (acrylic acid sodium) grafted carboxymethyl cellulose as a high performance polymer binder for silicon anode in lithium ion batteries

Liangming Wei,* Changxin Chen* & Hao Wei *

Key Laboratory for Thin Film and Microfabrication of the Ministry of Education, Department of Microelectronics and Nanoscience, School of Electronic Information and Electrical Engineering, Shanghai Jiao Tong University, 800 Dongchuan Road, Shanghai, 200240, China

* Correspondence and requests for materials should be addressed to L.M.W., C.C.C

or H. W (e-mail: lmwei@sjtu.edu.cn, haowei@sjtu.edu.cn , chen.c.x@sjtu.edu.cn)

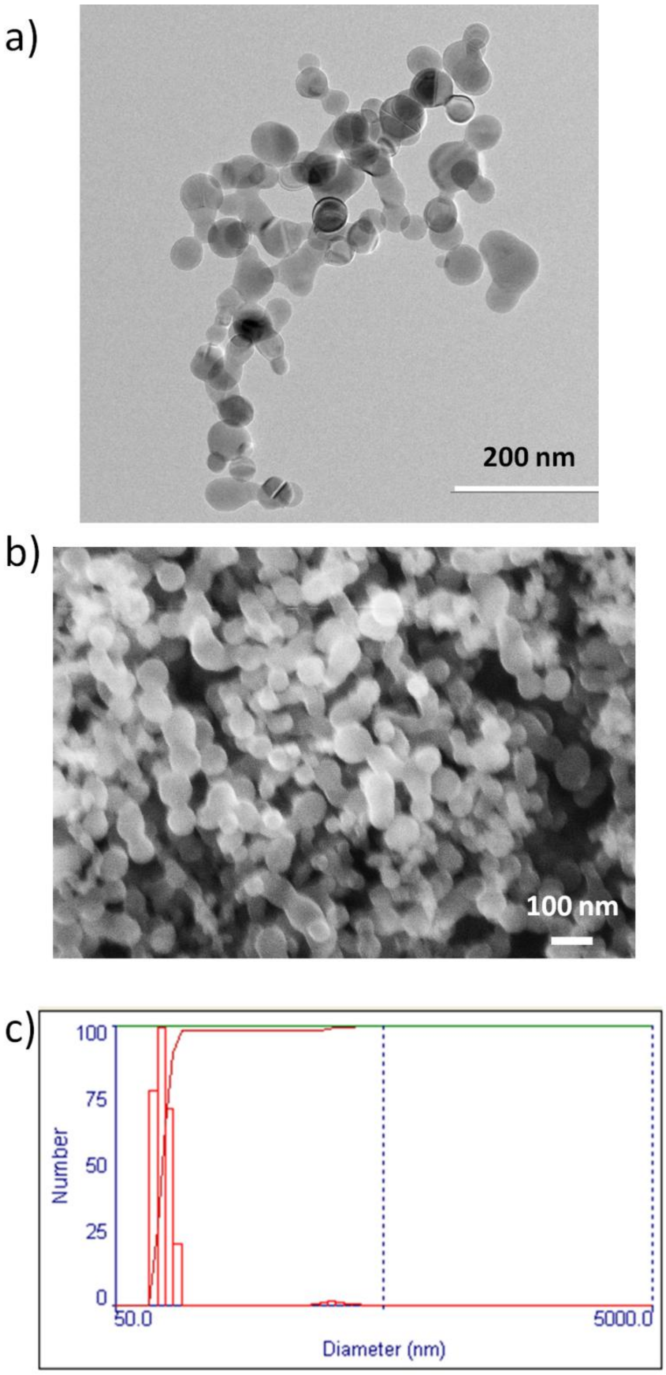

Figure S1. TEM (a), SEM (b) and the particle size analysis (c) of the pristine Si nanoparticles.

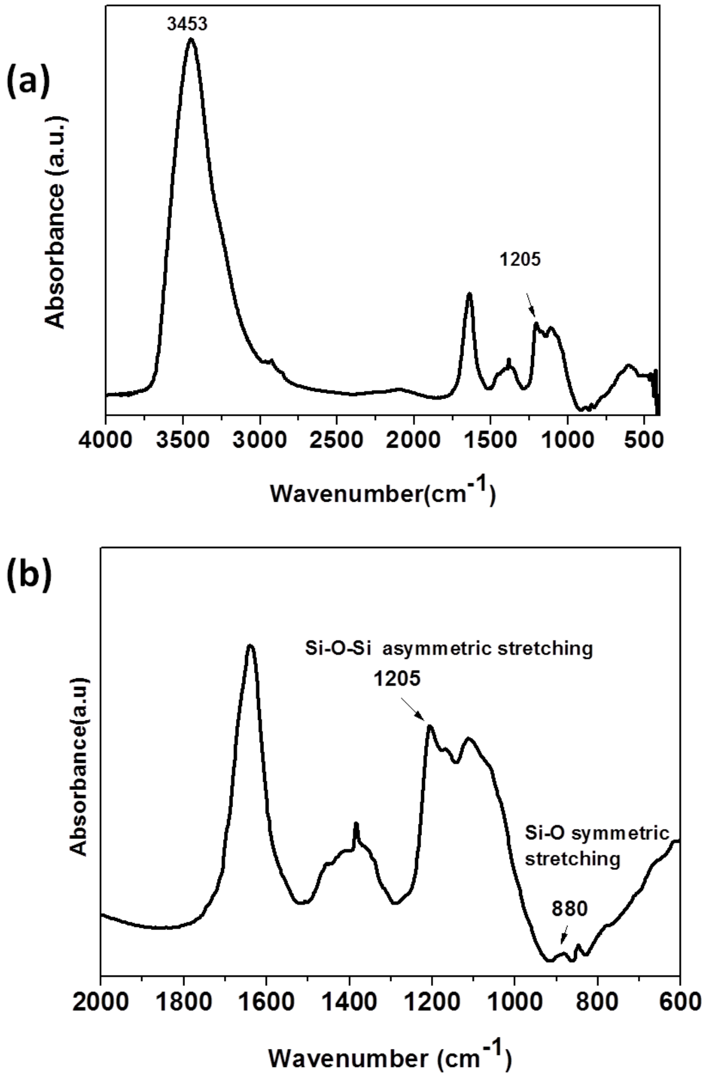

Figure S2. a) FTIR spectra of pure Si nanoparticles; b) highlighted some portions of the FTIR spectra.

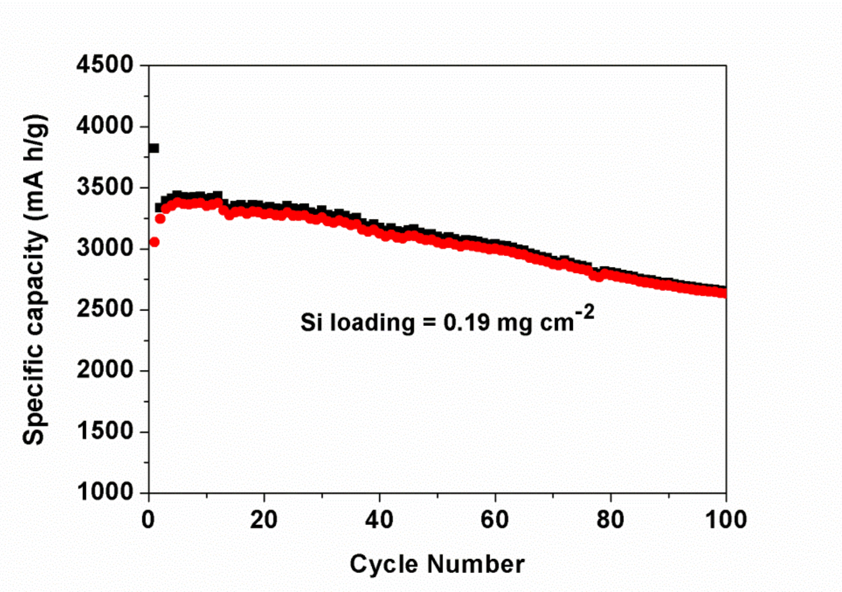

Figure S3. Cycling performance of Si anode with NaPAA-*g*-CMC binder. The mass loading of Si on Cu foil was 0.19 mg cm^{-2}.

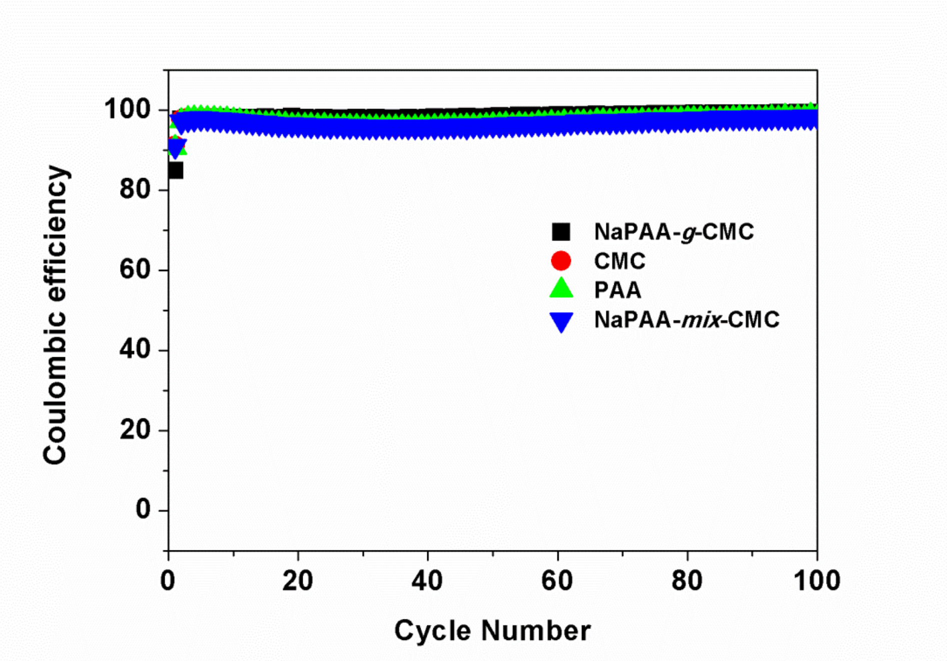

Figure S4. The coulombic efficiency of Si anodes with different binders.

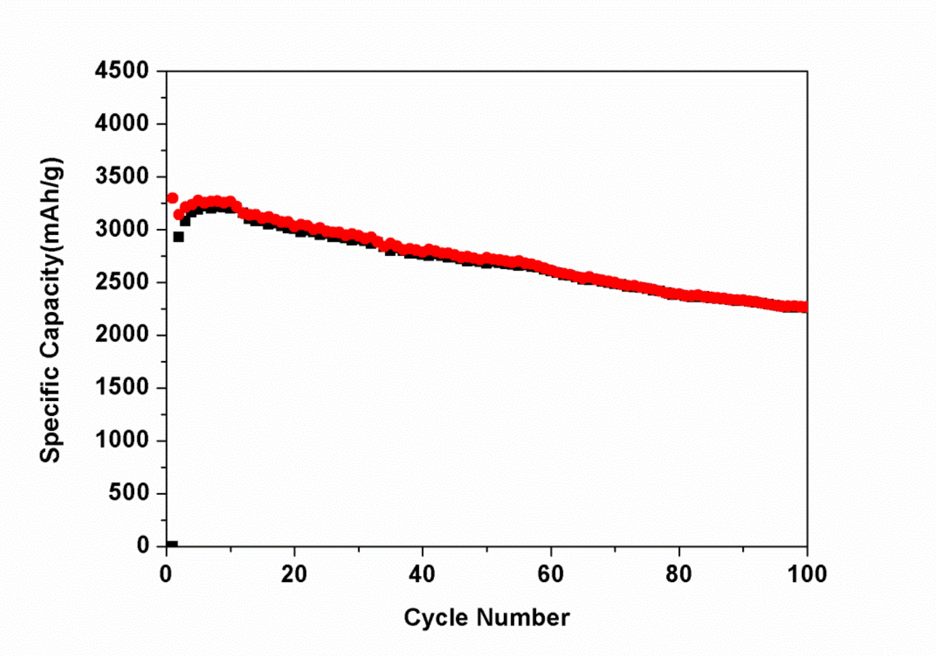

Figure S5. Cycling performance of Si anode with NaPAA-*g*-CMC binder after removal of persulfacte initiators.
